# Treatment of Aphthae

**Published:** 1910-06-15

**Authors:** 


### TREATMENT OF APHTHAE.

Aphthae when not associated with grave constitutional affections, such as aphthous stomatitis of aphthae fever, is considered as a mild affection which may be very painful to the subject and it may also become very annoying on account of its frequent reapparition in certain cases and with certain subjects.

The therapeutic of aphthae, according to M. Fargin-Fayolle, should be of the same nature as that of the soft chancre, that is, the immediate transformation of a specific ulceration into a commonly termed chancre sore which will readily heal with a treatment of silver-nitrate or chromic acid. But unfortunately, these agents will not generally destroy the ulceration in depth.

The electro-cautery may be used successfully as to the cure, but its application is indeed very painful.

On the contrary, the destruction of aphthae may be effected painless by the use of sulphuric acid of Nordhausen. Its application will give immediate relief, its mode of application only requires a little care.

Cauterization with two pencils, as advocated by M. Sabouraud in the local treatment of fungus or ulcerous scrogulo-dermatitis, applied to the treatment of aphthae by M. Fargin-Fayolle, gives good results.

This treatment consists in a double cauterization using the two pencils. The first by using the silver-nitrate pencil and the second with the metallic zinc pencil. The affected part is first touched with the silver-nitrate pencil, this area will soon take a whitish tint which will rapidly be followed by a black coloration as soon as the zinc pencil is applied. This is due to the double decomposition which takes place giving rise to formation of free nitric acid.

The zinc pencil, following this should be thoroughly washed.

The use of trichloracetic acid or of the acid nitrate of mercury may give excellent results, but their use is very dangerous.—*American Journal* for March.

